# Multivalent Anchoring and Oriented Display of Single-Domain Antibodies on Cellulose

**DOI:** 10.3390/s90705351

**Published:** 2009-07-07

**Authors:** Greg Hussack, Yan Luo, Linda Veldhuis, J. Christopher Hall, Jamshid Tanha, Roger MacKenzie

**Affiliations:** 1 Institute for Biological Sciences, National Research Council of Canada, 100 Sussex Drive, Ottawa, Ontario, Canada, K1A 0R6; 2 Department of Biochemistry, Microbiology and Immunology, University of Ottawa, Ottawa, Ontario, Canada, K1H 8M5; 3 Department of Environmental Biology, University of Guelph, Guelph, Ontario, Canada, N1G 2W1

**Keywords:** single-domain antibody, cellulose-binding module, bispecific, pentamer, *Staphylococcus aureus*, pathogen detection

## Abstract

Antibody engineering has allowed for the rapid generation of binding agents against virtually any antigen of interest, predominantly for therapeutic applications. Considerably less attention has been given to the development of diagnostic reagents and biosensors using engineered antibodies. Recently, we produced a novel pentavalent bispecific antibody (i.e., decabody) by pentamerizing two single-domain antibodies (sdAbs) through the verotoxin B subunit (VTB) and found both fusion partners to be functional. Using a similar approach, we have engineered a bispecific pentameric fusion protein consisting of five sdAbs and five cellulose-binding modules (CBMs) linked via VTB. To find an optimal design format, we constructed six bispecific pentamers consisting of three different CBMs, fused to the *Staphylococcus aureus-*specific human sdAb HVHP428, in both orientations. One bispecific pentamer, containing an N-terminal CBM9 and C-terminal HVHP428, was soluble, non-aggregating, and did not degrade upon storage at 4 °C for over six months. This molecule was dually functional as it bound to cellulose-based filters as well as *S. aureus* cells. When impregnated in cellulose filters, the bispecific pentamer recognized *S. aureus* cells in a flow-through detection assay. The ability of pentamerized CBMs to bind cellulose may form the basis of an immobilization platform for multivalent display of high-avidity binding reagents on cellulosic filters for sensing of pathogens, biomarkers and environmental pollutants.

## 1. Introduction

Single-domain antibodies (sdAbs), derived from the variable heavy (V_H_) and variable light (V_L_) domains of immunoglobulin G (IgG) [1], from the variable domain (i.e., V_H_H) of *Camelidae* species’ heavy-chain IgG [2,3] and more recently from the variable domain of shark immunoglobulin new antigen receptor (IgNAR) antibodies [4] have several characteristics that make them potential candidates for diagnostic and therapeutic applications [5,6]. These characteristics include: small size (14–15 kDa) and single domain nature [7], high solubility, high thermal and proteolytic stability [8–10], high target affinity (nM - pM range) [11], accessibility to cryptic target-antigens (Ag) [12] and high yields in bacterial and yeast expression systems [13,14]. The physical robustness and relatively low production cost of sdAbs make them logical antibody-based molecules for incorporation into immunosensors.

The generation of bispecific molecules, such as bispecific antibodies (bsAbs) which bind two distinct epitopes, has been one strategy to enhance the therapeutic potency of sdAbs and other antibody fragments such as Fabs (fragments antigen binding) and scFvs (single-chain fragments variable; reviewed in Holliger and Hudson [15]). Traditionally, bsAbs have been produced for the purpose of: (i) increasing the avidity of an Ab-Ag interaction by fusing two or more Abs which bind different epitopes on the same antigen [16] or (ii) activating innate and adaptive immune responses by fusing an Ab with specificity for effector cells to a second, target-specific Ab [17]. Other bispecific molecules containing antigen-specific antibody fragments fused to fragment crystallizable (Fc) regions have also been successfully produced [15]. Few authors, however, have examined the potential of bispecific molecules for diagnostic and biosensing applications. By replacing one of the antibodies in a bsAb with an immobilization domain, antibodies could conceivably be anchored to solid support matrices [6] for the specific capture and/or detection of food-, water-, or blood-borne pathogens, toxins, small molecules, or viruses. A molecule in which a sdAb is fused to an anchoring domain combines the many advantages of sdAbs, noted above, and the benefits of oriented immobilization of the detecting molecule on the biosensor surface. Simple adsorption or random coupling of antibody molecules to surfaces results in random orientation of the antibody molecule and can result in steric hindrance problems, antibody denaturation and, in the case of physical adsorption, loss of the antibody from the sensing surface. Collectively, this could compromise the effective antibody binding density and decrease biosensor sensitivity.

There is a need, particularly in the developing world, for inexpensive sensing devices, for clinical and environmental applications, that do not rely on sophisticated instrumentation. Cellulose is an attractive support matrix for the development of novel biosensing surfaces because of its chemical and physical stability, low cost, low nonspecific affinity for proteins and approval for human and therapeutic use [18]. Recently, paper-based microfluidic devices have been shown to perform well as low cost analytical systems for colourimetric bioassays [19,20]. In another cost-effective paper-based bioassay using gold nanoparticle colourimetric probes, the paper substrate was observed to provide a bright background and to protect the DNA-cross-linked nanoparticles used in the assay [21]. Cellulose-binding modules (CBMs), originally identified in *Trichoderma reesei* and *Cellulomonas fimi*, are the non-catalytic domains of cellulases and are responsible for anchoring and concentrating the enzymes on insoluble cellulose [22,23]. CBMs have high and specific affinity for a variety of soluble and insoluble celluloses, depending on their subfamily of origin (http://www.cazy.org/fam/acc_CBM.html). In the past, several CBMs have been fused to biologically active molecules such as enzymes, cytokines, and antibodies for various applications (see review by Shoseyov *et al.* [24]). To make CBM-antibody fusions a practical alternative to the covalent immobilization of antibodies for diagnostic applications, near irreversible anchoring of high-affinity antibodies is required. One possible approach to achieve this is to increase the avidity of cellulose-CBM and Ab-Ag interactions simultaneously through the multimerization of both CBM and Ab domains. Expression of sdAbs fused to verotoxin (VTB) has permitted the expression and assembly of pentameric antibodies with higher avidity and apparent affinities than monomeric versions of the same sdAbs [16].

**Figure 1.**
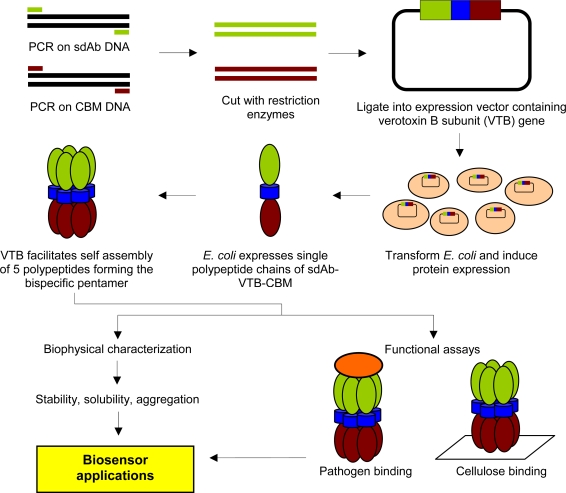
Overview of fusion protein construction and characterization.

Recently, a bispecific pentavalent antibody (i.e., decabody) was constructed by inserting the VTB gene between two single-domain antibodies capable of binding parathyroid hormone (PTH) [25]. Using a similar approach, our goal here was to engineer a pentameric, bispecific molecule that would bind cellulose, through five CBMs, and the human pathogen *Staphylococcus aureus*, through five copies of a human sdAb (referred to as HVHP428 [26]), which specifically recognizes cell-surface protein A (Figure 1). We designed six bispecific expression cassettes based on three CBM variants fused to either the N- or C- terminus of HVHP428 and characterized these fusion proteins. One format, consisting of an N-terminal CBM9 fusion (CBM9-VTB-HVHP428), was expressed and purified in soluble form from *E. coli* with only a small fraction prone to degradation. This bispecific pentamer was capable of binding to cellulose-based filters through the pentameric CBM and also retained its ability to agglutinate *S. aureus* cells through the pentameric sdAb. Furthermore, cellulose filters containing immobilized CBM9-VTB-HVHP428 were capable of detecting *S. aureus* cells to a limit of detection of 10^5^ cfu/mL (p = 0.05) when used in a vacuum flow-through assay.

## 2. Materials and Methods

### 2.1. Cell Lines and Plasmids

All bacterial cloning and expression was conducted in the pVT1 expression vector [25] using electrocompetent TG1 *Escherichia coli* from New England Biolabs (Mississauga, ON, Canada). For yeast cloning, constructs were assembled in the pPICZαA vector and expression was conducted in *Pichia pastoris* strain X-33 that was purchased from Invitrogen (Mississauga, ON, Canada). *S. aureus* (ATCC12598) was purchased from American Type Culture Collection (Manassas, VA, USA).

### 2.2. Cloning and Expression of sdAb-CBM Fusions

The human sdAb HVHP428 [26], which specifically recognizes *S. aureus* protein A, and three cellulose-binding modules: (i) CBM2a [27], (ii) CBM2a(m), a CBM2a mutant devoid of five putative N-glycosylation sites [28] and codon-optimized for *P. pastoris* (JCH, *unpublished data*), and (iii) CBM9 [29], were used. DNA encoding CBM2a and CBM9 were gifts from Dr. C.A. Haynes, University of British Columbia. A total of fourteen constructs were assembled with various combinations and orientations of HVHP428, CBM, or both. Of these, ten were expressed in either *E. coli* or *P. pastoris*. Briefly, CBM and sdAb genes were amplified by PCR and flanked with DNA encoding linker sequences and restriction sites *Bbs*I/*Apa*I or *Bsi*WI/*Apa*I for N-terminal fusions and *Bsp*EI/*Bam*HI for C-terminal fusions. PCR primers for gene amplification and introduction of restriction sites for ligation are summarized in Table 1. Three linkers designated L1, L2, and L3, which separate the CBM, VTB, and HVHP428 domains, were the same linkers as those used for a clone in a previously described bispecific pentamer [25]. Amplified insert DNA was digested with the corresponding restriction enzymes and ligated into a similarly digested pVT1 vector. One clone, HVHP428-VTB-CBM2a(m), which contained a CBM devoid of the putative N-glycosylation site A-X-T/S (X = any amino acid except P), was subcloned into the pPICZαA expression vector from pVT1 and transformed into chemically competent *P. pastoris* X-33 according to the manufacturer’s instructions (Invitrogen). Monospecific and bispecific pentamers were expressed in *E. coli* as described [16]. Protein expression in *P. pastoris* was induced with 0.5% methanol for four days and proteins were purified according to the manufacturer’s instructions (Invitrogen). Expression of recombinant proteins was monitored by Western blotting using anti-His_6_ IgG (GE Healthcare, Baie-d’Urfé, QC, Canada) for detection (data not shown).

### 2.3. Purification of Recombinant Proteins and Size Exclusion Chromatography

*E. coli* cells were harvested at 12,000 rpm × 20 min, supernatant removed, and the pellets processed by total cell lysis [25]. Soluble fractions were dialyzed into immobilized metal affinity chromatography (IMAC) buffer A (10 mM HEPES buffer pH 7.0, 500 mM NaCl), sterile filtered, and protein was purified by IMAC using 5 mL HiTrap™ Chelating HP IMAC columns (GE Healthcare), under the control of an ÄKTA™-FPLC (GE Healthcare). A step-wise gradient of 500 mM imidazole in the above buffer was used for protein elution. Proteins were stored at 4 °C. Insoluble proteins found in inclusion bodies were not processed further. Size exclusion chromatography was performed on all purified proteins with a Superdex 200™ column under the control of an ÄKTA™-FPLC, as described [25]. For *P. pastoris*, recombinant proteins were secreted into the culture medium, cells were harvested and the clarified supernatant was loaded onto a HiTrap™ IMAC column and eluted as described above.

### 2.4. Microagglutination Assays

A microagglutination assay was performed to determine whether CBM9-VTB-HVHP428 retained its ability to bind and agglutinate *S. aureus* cells. Fifty microlitre aliquots of gamma-irradiated *S. aureus* cells (1 × 10^8^ cfu/mL) were added to 96-well microtitre plates. Proteins (CBM9-VTB-HVHP428, HVHP428-VTB, and HVLP335-VTB [26]) were serially diluted 2-fold in MES buffer (30 mM MES pH 6.0, 70 mM NaCl) in 11 wells. The last well contained MES buffer and *S. aureus*. Starting concentrations were 600 nM for HVHP428-VTB, CBM9-VTB-HVHP428, and VHLP335-VTB control. After addition of cells and proteins, the mixtures were incubated overnight (18 h) at 4 °C and scored visually for agglutination.

### 2.5. Cellulose Binding Assays

To determine if CBM9-VTB-HVHP428 binds to cellulose, 2-fold serial dilutions of purified protein were spotted onto Whatman No. 5 filter paper (GE Healthcare) starting at 800 nM with a 96-well Bio-Dot vacuum manifold from Bio-Rad (Hercules, CA, USA). All protein dilutions were prepared in PBS. The filter paper was washed 3x with PBS and blocked for 1 h at room temperature with 5% BSA (w/v) in PBS. Protein A conjugated with alkaline phosphatase (AP) was purchased from Pierce (Rockford, IL, USA) and applied to the paper using the vacuum manifold (100 μL of a 1:5,000 dilution) followed by the addition of AP substrate (Bio-Rad) to detect CBM9-VTB-HVHP428 binding. HVHP428-VTB was applied to the filter paper with a starting concentration of 800 nM and served as a negative control.

### 2.6 Detection of S. aureus with CBM9-VTB-HVHP428

Circular cellulose discs (6 mm diameter, 20–25 μm pore size) were prepared from Whatman 451 filter paper (GE Healthcare) using a standard hole-punch. Discs were pre-wetted with PBS and placed in the 96-well Bio-Dot vacuum manifold (Bio-Rad). All experiments were conducted at 20 °C. To “load” the filters, 5 μL of CBM9-VTB-HVHP428 (∼200 μg/mL) or 5 μL of VTB-HVHP428 (∼100 μg/mL) diluted in PBS were applied to the circular cellulose discs without suction, incubated for 20 min, and then pulled through under vacuum pressure. “Loaded” and untreated discs were blocked with 300 μL of 3% (w/v) skim milk in PBS and washed 3x with 300 μL PBS-0.05% (v/v) Tween 20 (PBST). Five microliters of serially diluted gamma-irradiated *S. aureus* (starting at 1 × 10^8^ cfu/mL) were applied to the cellulose filters without suction, incubated for 30 min, and pulled through the filters under vacuum pressure. Discs were washed 3x with PBST, incubated with anti-protein A-HRP IgG (Invitrogen; 1:5,000 dilution, 20 min incubation), and washed an additional 3x with PBST followed by 2x with PBS. For detection of binding, discs were transferred to a 96-well microtiter plate, and submersed in 150 μL of peroxidase substrate from Mandel Scientific (Guelph, ON, Canada) for 45 min. The reaction was stopped by the addition of 150 μL 10% H_2_SO_4_ to each well. Discs were removed from the plate and well contents were read at 450 nm using a Bio-Rad plate reader.

## 3. Results

### 3.1. Expression and Characterization of CBM-sdAb Fusion Proteins

**Figure 2.**
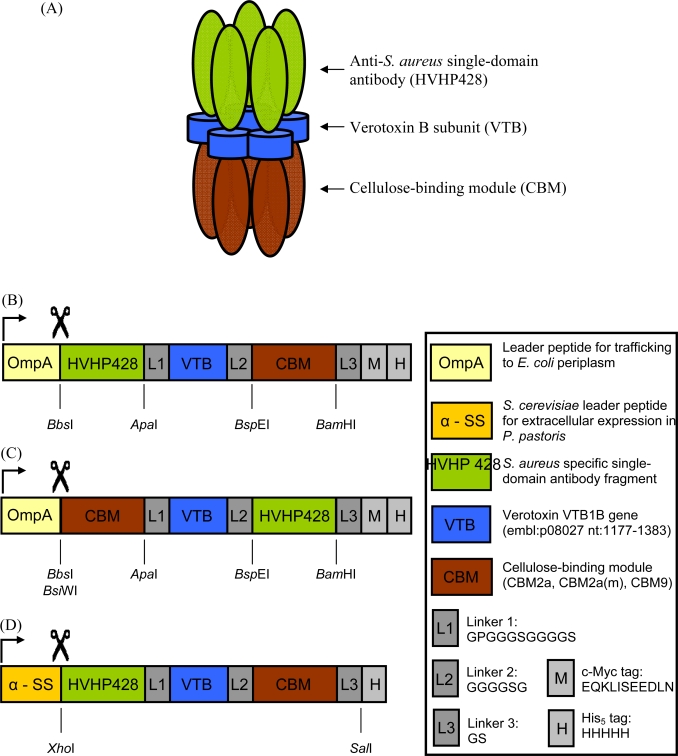
Assembly of bispecific pentamers. (A) Schematic representation of a bispecific pentamer displaying five human sdAbs fused to five CBMs through the verotoxin subunit (VTB). (B-D) Schematic representation of the coding sequences assembled and expressed in pVT1 (B, C) or pPICZαA (D).

**Figure 3.**
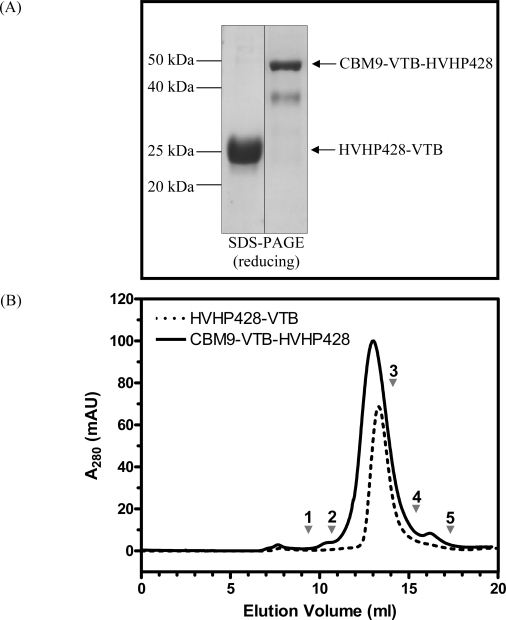
Analysis of purified monospecific and bispecific pentamers. (A) SDS-PAGE (reducing conditions, ∼3 μg protein per lane) demonstrating the stability of purified proteins which had been stored at 4 °C for six months. The reducing conditions separated the pentamerized molecules into five single polypeptide chains. The expected masses of monospecific HVHP428-VTB and bispecific CBM9-VTB-HVHP428 in an SDS-PAGE gel are 24.4 kDa and 45.7 kDa, respectively. (B) Size exclusion chromatography using a Superdex 200™ column revealed both HVHP428-VTB and CBM9-VTB-HVHP428 were non-aggregating since a single, homogeneous peak was observed for each within the expected mass range. The bispecific CBM9-VTB-HVHP428 pentamer eluted later than expected. Protein standards are indicated with arrows. 1: thyroglobulin (M_r_ 669,000); 2: ferritin (M_r_ 440,000); 3: BSA (M_r_ 67,000); 4: beta-lactoglobulin (M_r_ 35,000); 5: ribonuclease A (M_r_ 13,700).

Previously, we reported the assembly of pentameric single-domain antibodies (sdAbs) by fusing a parathyroid hormone peptide-specific V_H_H to the C-terminus of VTB [16] and more recently the generation of bispecific pentameric sdAbs (i.e., decabodies) by fusing two sdAbs through the VTB gene [25]. The pentameric fusions increased overall apparent affinities towards the parathyroid hormone peptide antigen by 10^3^ to 10^4^ fold [16] and the bispecific pentamer resulted in a molecule with ten functional antibodies capable of antigen binding [25]. To develop a platform for immobilizing pentameric sdAbs on solid cellulose supports, we produced several cellulose-binding module (CBM)–sdAb fusion constructs which contained an internal VTB homopentamer for assembly (Figure 2). A total of eight monospecific (i.e., CBM or sdAb) and 6 bispecific (i.e., CBM + sdAb) constructs were assembled and characterized based on soluble expression in *E. coli*, expression yield, as well as susceptibility to both aggregation and degradation (Table 2). For all constructs involving sdAbs, the anti-*S. aureus* human sdAb HVHP428 [26] was used. N-terminal fusions of HVHP428 to VTB (HVHP428-VTB) were produced as soluble, non-aggregating, monospecific pentamers (Figure 3). N-terminal CBM2a-VTB expressed in soluble fractions, whereas N-terminal CBM2a(m)-VTB was found in inclusion bodies. Conversely, C-terminal VTB-CBM2a(m) was found to be soluble. Three bispecific combinations containing C-terminal CBM2a(m), CBM2a, and CBM9 did not express at all. One of these fusion proteins, HVHP428-VTB-CBM2a(m) was subcloned into the pPICZαA vector and was found to express in *P. pastoris*, but not explored further. Three other bispecific clones containing N-terminal CBMs were found to be soluble when expressed in *E. coli* and were characterized further. Bispecific pentamers containing N-terminal CBM2a or CBM2a(m) were prone to aggregation and degraded. However, one bispecific pentamer containing an N-terminal CBM9 showed no aggregation tendency and only minor degradation (Figure 3), with an expression yield of 7.8 mg/L of bacterial culture.

### 3.2. Functional Characterization of CBM9-VTB-HVHP428 Bispecific Pentamer

**Figure 4.**
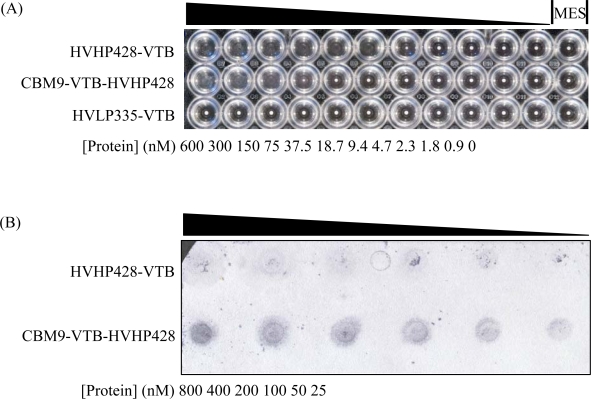
Functional characterization of CBM9-VTB-HVHP428. (A) Agglutination of *S. aureus* (wells without small white dots in the middle) was seen for CBM9-VTB-HVHP428 with an end limit of 150 nM compared to an end limit of 18.7 nM for the positive control, HVHP428-VTB. No agglutination was seen for the negative control, HVLP335-VTB [26]. (B) Binding of CBM9-VTB-HVHP428 to cellulose. Serial dilutions of purified CBM9-VTB-HVHP428 and HVHP428-VTB were applied to Whatman No. 5 filter paper using a vacuum manifold. Binding was detected by incubating the paper with alkaline phosphatase-labeled protein A.

To determine whether CBM9-VTB-HVHP428 was capable of specifically binding *S. aureus* cells and cellulose, two assays were performed. Microagglutination of *S. aureus* with CBM9-VTB-HVHP428 confirmed that HVHP428 retained its ability to bind protein A when assembled as part of the bispecific pentamer (Figure 4A), albeit not as effectively as the monospecific pentamer HVHP428-VTB. The lowest concentration of CBM9-VTB-HVHP428 to agglutinate *S. aureus* was 150 nM compared to 18.7 nM for HVHP428-VTB. The monospecific pentamer control, HVHP335-VTB, which does not recognize *S. aureus* [26], did not agglutinate cells at any of the concentrations tested. In the second assay, mono- and bispecific pentamers were added to Whatman No. 5 filter paper using a vacuum manifold and probed with alkaline phosphatase labeled protein A (Figure 4B). We conclude that CBM9-VTB-HVHP428 specifically bound to cellulose paper because only a small fraction of HVHP428-VTB, when used as a control, bound non-specifically to the paper.

### 3.3. Detection of S. aureus with CBM9-VTB-HVHP428 Impregnated Filters

**Figure 5.**
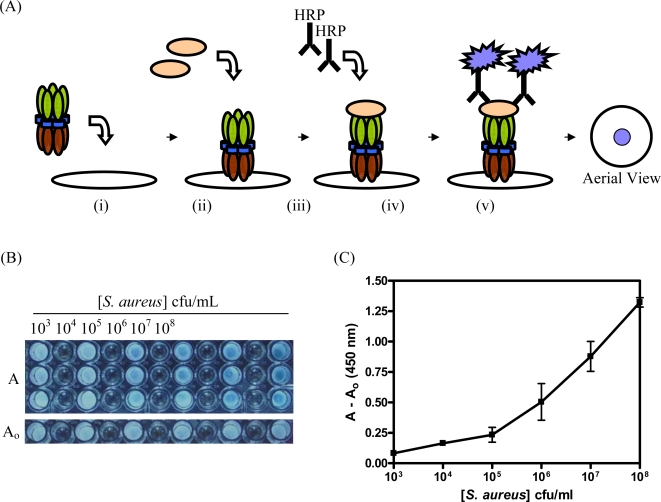
Detection of *S. aureus* with immobilized CBM9-VTB-HVHP428. (A) Schematic representation of the CBM9-VTB-HVHP428-based biosensor. (i) Loading of bispecific pentamer onto the filter, (ii) *S. aureus* cells passed over the filter, (iii) HRP labeled anti-protein A IgG passed over the filter, (iv) the filter moved to 96 well plates, substrate applied, and the absorbance read at 450 nm, (v) aerial view of filter after substrate. (B) Photograph of experimental filter discs after removal of HRP substrate from a 96 well plate. (C) Plot of absorbance A (A_450_ of CBM9-VTB-HVHP428 impregnated discs) - A_o_ (A_450_ of control discs with HVHP428-VTB) *versus* the number of *S. aureus* colony forming units (cfu) applied to each disc. Limit of detection = 10^5^ cfu/mL (p = 0.05). Error bars represent standard error of the mean (SEM) based on A_450_ values from two independent experiments with three replicates in each.

To determine if CBM9-VTB-HVHP428 could be used for the detection of *S. aureus*, cellulose filter discs were impregnated with the molecule and *S. aureus* cells were passed through the discs (Figure 5A) with the assistance of a vacuum manifold. HVHP428-VTB-treated and blank filter discs (i.e., no protein) were used as negative controls. Following probing with anti-protein A-HRP IgG, it was determined that CBM9-VTB-HVHP428 was capable of readily detecting 10^5^ cfu/mL of *S. aureus* cells (p = 0.05) and as few as 10^4^ cfu/mL (p = 0.1), when compared to control discs containing monospecific pentamer (Figure 5B and 5C). Filters alone (i.e., containing no applied protein) did not bind *S. aureus* at any of the concentrations tested (data not shown). Based on these results, bispecific fusion proteins containing five CBM domains and five sdAbs appear to retain dual functionality when immobilized on cellulose supports.

## 4. Discussion

In this work, we set out to develop an immobilization platform for the oriented display of sdAbs with high valency and apply this platform for pathogen biosensing. We demonstrated that it is possible to make a bispecific fusion protein consisting of a CBM for immobilization on cellulose joined to a human sdAb for antigen binding all linked via the VTB subunit. Furthermore, five of these bispecific polypeptide chains assemble to make a bispecific pentamer with extremely high antigen and cellulose affinity due to the avidity conferred by its pentameric structure. This work is the first to report the expression of functional pentameric CBMs and the first to demonstrate fusion of human sdAbs to CBMs.

Compared to chemical cross-linking of antibodies to solid supports where random orientation and steric hindrance can decrease the activity of immobilized ligand [30], CBMs are thought to orient their fusion partners away from the immobilization surface, allowing for the biologically active fusion partner to access its target substrate or antigen. In this study, we tested three CBMs as either N- or C- terminal fusions to HVHP428 and found one molecule, CBM9-VTB-HVHP428, which expressed as a soluble, non-aggregating bispecific pentamer in *E. coli*. Several of our other bispecific pentamers containing CBMs and HVHP428 either did not express in *E. coli*, were insoluble, or were prone to both aggregation and degradation. However, we found that fusions containing N-terminal CBMs were soluble in *E. coli*. These results are in agreement with the literature where more than twelve functional fusion proteins containing an N-terminal CBM9 affinity tags have been reported [31]. Interestingly, we found that fusions containing C-terminal CBMs did not express well in *E. coli*. While no C-terminal CBM9 fusion proteins have been reported, most CBM2a (formerly CBD_Cex_) fusion proteins described by other researchers contain the CBM at the C-terminus [27,32].

The poor expression of C-terminal-containing CBM fusion proteins was somewhat surprising since CBMs are naturally found at different positions within the cellulase polypeptide chain, i.e. at the N-terminal, C-terminal, or between these two termini [24]. Our results indicate that the optimal CBM orientation in the recombinant fusion proteins produced here differs from the orientation of the same CBM found in nature. This is likely due to the influence of the fusion partner on the global conformation of the fusion protein, as non-optimal orientations may expose internal hydrophobic residues (causing aggregation and poor solubility), or may negatively affect the activity of one or both of the fusion partners, as seen in our agglutination assay results (Figure 4A). Most CBMs rely on surface-exposed aromatic residues, such as tryptophan, for cellulose binding [27]. The pentamerization of CBM9 within the CBM9-VTB-HVHP428 molecule did not appear to sterically hinder its interaction with cellulose, as some or all of the external aromatic residues putatively responsible for binding must be sufficiently close enough to the sugar residues to form hydrogen bonds. Previously, Linder *et al*. [33] produced a double cellulose-binding domain fusion protein consisting of two CBMs from *Trichoderma reesei* and found the fusion to bind cellulose with higher affinity than either of the CBMs alone. The authors also concluded the double CBMs did not sterically hinder each others’ access to the cellulose surface. Elsewhere, an anti-azo red dye llama V_H_H single-domain antibody fusion protein containing two CBMs was found to adhere to cellulose stronger than V_H_H fusions containing a single CBM [35]. Based on the ability of CBM9-VTB-HVHP428 to bind cellulose in our assay (Figure 4B), it is assumed that the some or all of the five CBM domains have free access to cellulose surfaces.

In this study we also demonstrated that five HVHP428 sdAbs retained their ability to bind *S. aureus* antigen when expressed as part of the bispecific pentamer. This was not surprising since production of pentabody [16] and decabody [25] formats using llama sdAbs resulted in functionally active antibodies with higher overall affinities than their monomeric counterparts. Specifically, CBM9-VTB-HVHP428 was capable of binding *S. aureus* in solution and when immobilized on cellulose filters. However, the lowest concentration of CBM9-VTB-HVHP428 to agglutinate *S. aureus* in the microagglutination assay was significantly higher than that of the control HVHP428 pentamer, which does not contain CBM9, indicating the CBM9 fusion may have some detrimental effect on sdAb activity. When CBM9-VTB-HVHP428 was immobilized on cellulosic filters, HVHP428 retained its ability to bind *S. aureus* cells. This suggests HVHP428 was not buried in the filter; rather, it was orientated to permit binding to *S. aureus*. Collectively, our results indicate that both pentamerized domains (i.e., sdAb and CBM) retained dual functionality. These results agree with previous work which showed that llama V_H_H single-domain antibodies retain functionality when fused to CBMs [34,35].

The use of recombinant single-domain antibody fragments (i.e., V_H_H, V_H_, V_L_), when fused to immobilization domains such as CBMs, is a low-cost biosensing platform that maintains high target specificity and affinity. The single-domain nature of the antibodies used in this study was likely critical to the successful formation of a functional, bispecific pentamers. As such, one would expect more complex recombinant antibody formats (e.g., scFvs) to perform poorly in these multivalent, antigen-binding structures due to their vulnerability to intermolecular domain association.

## 5. Conclusions

In summary, the bispecific molecule produced in this study was simultaneously capable of immobilization on cellulose and oriented display of high-avidity human sdAbs for antigen capture. We have demonstrated a rapid, near real-time biosensing assay for pathogen detection by immobilizing this sdAb-CBM fusion protein on a cheap and abundant support matrix, cellulose, and detected pathogen capture with commercially available assay reagents. The strong affinity attachment of the biorecognition molecule to the sensing surface is preferable to physical adsorption because of the oriented display and the essentially irreversible nature of the attachment. These are important features of any bioactive paper-based sensor. We envision that these bispecific pentamers may form the basis of an immobilization platform for displaying high valency reagents, such as engineered antibodies and enzymes, on paper for applications ranging from blood filtration, to water purification, and pathogen detection. Given the demonstrated feasibility of printing microfluidic channels on paper surfaces [19,20], it is easy to envision a device in which the test sample is drawn over a surface on which the bispecific molecule is immobilized. Following capture of the target pathogen or molecule, a suitable amount of the detection reagent could be drawn over the biosensing area on the paper.

## Figures and Tables

**Table 1.** Oligonucleotides used in the assembly of expression constructs.

**Construct**	**Oligonucleotide sequence** (5′ → 3′, enzyme sites underlined)	**RE**
1-For	GTTGCGCAGGCCGTCTTCCAGCTGCAGCTGCAGGAGT	*Bbs*I
1-Rev	GGAGCCGCCGCCGGGCCCTGAGGAGACGGTGACCA	*Apa*I
2-For	GGTGGGTCCGGACAGCTGCAGCTGCAGGAG	*Bsp*EI
2-Rev	AGTTTTTGTTCGGATCCTGAGGAGACGGTGACCA	*Bam*HI
3-For	GGTGGGTCCGGATCTGGTCCGGCGGGTTGTC	*Bsp*EI
3-Rev	AGTTTTTGTTCGGATCCACCAACGGTACACGGGGCAC	*Bam*HI
4-For	GGTGGGTCCGGAAGCGGCCCGGCCGGGTG	*Bsp*EI
4-Rev	AGTTTTTGTTCGGATCCGCCGACCGTGCAGGGCGTG	*Bam*HI
5-For	GGTGGGTCCGGAATGACTAGCGGAATAATGGTAG	*Bsp*EI
5-Rev	AGTTTTTGTTCGGATCCAAAGCTTGATGAGCCTGAGG	*Bam*HI
6-For	GTTGCGCAGGCCGTCTTCTCTGGTCCGGCGGGTTGTC	*Bbs*I
6-Rev	GGAGCCGCCGCCGGGCCCACCAACGGTACACGGGGCAC	*Apa*I
7-For	GTTGCGCAGGCCGTCTTCAGCGGCCCGGCCGGGTG	*Bbs*I
7-Rev	GGAGCCGCCGCCGGGCCCGCCGACCGTGCAGGGCGTG	*Apa*I
8-For	GGCCGTCTTCGTACGAATGACTAGCGGAATAATGGTAG	*Bsi*WI
8-Rev	GGAGCCGCCGCCGGGCCCAAAGCTTGATGAGCCTGAG	*Apa*I
9-For (pPICZαA)	TTTTGTCTCGAGAAAAGAGAGGCTGAAGCTCAGCTGCAGCTGCAGGAGTCG	*Xho*I
9-Rev (pPICZαA)	TTTTGTGTCGACCTATCAATGGTGATGGTGATGGTTCAGATC	*Sal*I

**Table 2.** Summary of constructs, expression profiles, and purified proteins.

**Construct (No./Description)**	**Expression**		**Purified Protein**		

	***E. coli***	***P. pastoris***	**Yield (mg/L)**	**Aggregation^a^**	**Degradation^b^**
**Monospecifc**					

1 HVHP428-VTB	S		15.7	No	No
2 VTB-HVHP428					
3 VTB-CBM2a(m)	S		NA	NA	NA
4 VTB-CBM2a					
5 VTB-CBM9					
6 CBM2a(m)-VTB	IB				
7 CBM2a-VTB	S		NA	NA	NA
8 CBM9-VTB					

**Bispecific**					

9 HVHP428-VTB-CBM2a(m)	NE	S	NA	NA	NA
10 HVHP428-VTB-CBM2a	NE				
11 HVHP428-VTB-CBM9	NE				
12 CBM2a(m)-VTB-VHV28	S		Low	Yes	Yes
13 CBM2a-VTB-HVHP428	S		3.8	Yes	Yes
14 CBM9-VTB-HVHP428	S		7.8	No	Low

S = soluble, IB = inclusion bodies, NE = no expression, NA = not attempted.

a Determined by size exclusion chromatography.

b Determined by SDS-PAGE.

## References

[1] Ward E.S., Güssow D., Griffiths A.D., Jones P.T., Winter G. (1989). Binding activities of a repertoire of single immunoglobulin variable domains secreted from *Escherichia coli*. Nature.

[2] Hamers-Casterman C., Atarhouch T., Muyldermans S., Robinson G., Hamers C., Songa E.B., Bendahman N., Hamers R. (1993). Naturally occurring antibodies devoid of light chains. Nature.

[3] Arbabi-Ghahroudi M., Desmyter A., Wyns L., Hamers R., Muyldermans S. (1997). Selection and identification of single domain antibody fragments from camel heavy-chain antibodies. FEBS Lett.

[4] Nuttall S.D., Krishnan U.V., Hattarki M., De Gori R., Irving R.A., Hudson P.J. (2001). Isolation of the new antigen receptor from wobbegong sharks, and use as a scaffold for the display of protein loop libraries. Mol. Immunol.

[5] Goldman E.R., Liu J.L., Bernstein R.D., Swain M.D., Mitchell S.Q., Anderson G.P. (2009). Ricin detection using phage displayed single domain antibodies. Sensors.

[6] Saerens D., Huang L., Bonroy K., Muyldermans S. (2008). Antibody fragments as probe in biosensor development. Sensors.

[7] Tanha J., Xu P., Chen Z., Ni F., Kaplan H., Narang S.A., MacKenzie C.R. (2001). Optimal design features of camelized human single-domain antibody libraries. J. Biol. Chem.

[8] van der Linden R.H., Frenken L.G., de Geus B., Harmsen M.M., Ruuls R.C., Stok W., de Ron L., Wilson S., Davis P., Verrips C.T. (1999). Comparison of physical chemical properties of llama VHH antibody fragments and mouse monoclonal antibodies. Biochim. Biophys. Acta.

[9] Dumoulin M., Conrath K., van Meirhaeghe A., Meersman F., Heremans K., Frenken L.G., Muyldermans S., Wyns L., Matagne A. (2002). Single-domain antibody fragments with high conformational stability. Protein Sci.

[10] Harmsen M.M., van Solt C.B., van Bemmel A.M., Niewold T.A., van Zijderveld F.G. (2006). Selection and optimization of proteolytically stable llama single-domain antibody fragments for oral immunotherapy. Appl. Microbiol. Biotechnol.

[11] Koide A., Tereshko V., Uysal S., Margalef K., Kossiakoff A.A., Koide S. (2007). Exploring the capacity of minimalist protein interfaces: interface energetics and affinity maturation to picomolar KD of a single-domain antibody with a flat paratope. J. Mol. Biol.

[12] Stijlemans B., Conrath K., Cortez-Retamozo V., van Xong H., Wyns L., Senter P., Revets H., De Baetselier P., Muyldermans S., Magez S. (2004). Efficient targeting of conserved cryptic epitopes of infectious agents by single domain antibodies. African trypanosomes as paradigm. J. Biol. Chem.

[13] Arbabi-Ghahroudi M., Tanha J., MacKenzie R. (2005). Prokaryotic expression of antibodies. Cancer Metastasis Rev.

[14] Frenken L.G., van der Linden R.H., Hermans P.W., Bos J.W., Ruuls R.C., de Geus B., Verrips C.T. (2000). Isolation of antigen specific llama VHH antibody fragments and their high level secretion by *Saccharomyces cerevisiae*. J. Biotechnol.

[15] Holliger P., Hudson P.J. (2005). Engineered antibody fragments and the rise of single domains. Nat. Biotechnol.

[16] Zhang J., Tanha J., Hirama T., Khieu N.H., To R., Tong-Sevinc H., Stone E., Brisson J.-R., MacKenzie C.R. (2004). Pentamerization of single-domain antibodies from phage libraries: a novel strategy for the rapid generation of high-avidity antibody reagents. J. Mol. Biol.

[17] De Jonge J., Heirman C., de Veerman M., van Meirvenne S., Moser M., Leo O., Thielemans K. (1998). *In vivo* retargeting of T cell effector function by recombinant bispecific single chain Fv (anti-CD3 × anti-idiotype) induces long-term survival in the murine BCL1 lymphoma model. J. Immunol.

[18] Rodriguez B., Kavoosi M., Koska J., Creagh A.L., Kilburn D.G., Haynes C.A. (2004). Inexpensive and generic affinity purification of recombinant proteins using a family 2a CBM fusion tag. Biotechnol. Prog.

[19] Martinez A.W., Phillips S.T., Carrilho E., Thomas S.W., Sindi H., Whitesides G.M. (2008). Simple telemedicine for developing regions: camera phones and paper-based microfluidic devices for real-time, off-site diagnosis. Anal. Chem.

[20] Li X., Tian J., Nguyen T., Shen W. (2008). Paper-based microfluidic devices by plasma treatment. Anal. Chem.

[21] Zhao W., Ali M.M., Aguirre S.D., Brook M.A., Li Y. (2008). Paper-based bioassays using gold nanoparticle colorimetric probes. Anal. Chem.

[22] van Tilbeurgh H., Tomme P., Claeyssens M., Bhikhabhai R., Pettersson G. (1986). Limited proteolysis of the cellobiohydrolase I from *Trichoderma reesei*. Separation of functional domains. FEBS Lett.

[23] Gilkes N.R., Warren R.A., Miller R.C., Kilburn D.G. (1988). Precise excision of the cellulose binding domains from two *Cellulomonas fimi* cellulases by a homologous protease and the effect on catalysis. J. Biol. Chem.

[24] Shoseyov O., Shani Z., Levy I. (2006). Carbohydrate binding modules: biochemical properties and novel applications. Microbiol. Mol. Biol. Rev.

[25] Stone E., Hirama T., Tanha J., Tong-Sevinc H., Li S., MacKenzie C.R., Zhang J. (2007). The assembly of single domain antibodies into bispecific decavalent molecules. J. Immunol. Methods.

[26] To R., Hirama T., Arbabi-Ghahroudi M., MacKenzie R., Wang P., Xu P., Ni F., Tanha J. (2005). Isolation of monomeric human V(H)s by a phage selection. J. Biol. Chem.

[27] McLean B.W., Bray M.R., Boraston A.B., Gilkes N.R., Haynes C.A., Kilburn D.G. (2000). Analysis of binding of the family 2a carbohydrate-binding module from *Cellulomonas fimi* xylanase 10A to cellulose: specificity and identification of functionally important amino acid residues. Protein Eng.

[28] Boraston A.B., Warren R.A.J., Kilburn D.G. (2001). Glycosylation by *Pichia pastoris* decreases the affinity of a family 2a carbohydrate-binding module from *Cellulomonas fimi*: a functional and mutational analysis. Biochem. J.

[29] Kavoosi E., Meijer J., Kwan E., Creagh A.L., Kilburn D.G., Haynes C.A. (2004). Inexpensive one-step purification of polypeptides expressed in *Escherichia coli* as fusions with the family 9 carbohydrate-binding module of xylanase 10A from *T. maritima*. J. Chromatogr. B.

[30] Wimalasena R.L, Wilson G.S. (1991). Factors affecting the specific activity of immobilized antibodies and their biologically active fragments. J. Chromatogr.

[31] Kavoosi M., Sanaie N., Dismer F., Hubbuch J., Kilburn D.G., Haynes C.A. (2007). A novel two-zone protein uptake model for affinity chromatography and its application to the description of elution band profiles fused to a family 9 cellulose binding module affinity tag. J. Chromatogr. A.

[32] Ramirez C., Fung J., Miller R.C., Antony R., Warren J., Kilburn D.G. (1993). A bifunctional affinity linker to couple antibodies to cellulose. Biotechnology.

[33] Linder M., Salovuori I., Ruohonen L., Teeri T.T. (1996). Characterization of a double cellulose-binding domain. Synergistic high affinity binding to crystalline cellulose. J. Biol. Chem.

[34] Pangu G., Johnston E., Petkov J., Parry N., Leach M., Hammer D.A. (2007). Targeted particulate adhesion to cellulose surfaces mediated by bifunctional fusion proteins. Langmuir.

[35] Lewis W., Keshavarz-Moore E., Windust J., Bushell D., Parry N. (2006). Construction and evaluation of novel fusion proteins for targeted delivery of micro particles to cellulose surfaces. Biotechnol. Bioeng.

